# Differential responses of stressful elements to predatory exposure in behavior-lateralized mice

**DOI:** 10.1186/s12993-018-0144-9

**Published:** 2018-06-08

**Authors:** Jiacai Yang, Lin Zhang, Jian-ping Dai, Jun Zeng, Xiao-xuan Chen, Ze-feng Xie, Kang-sheng Li, Yun Su

**Affiliations:** 1 Department of Microbiology & Immunology, Shantou University Medical College, 22 Xinling Road, Shantou, 515041 Guangdong People’s Republic of China; 2 First Affiliated Hospital of Shantou University Medical College, Shantou, 515041 Guangdong People’s Republic of China

**Keywords:** Predator, Stress, Behavior lateralization, HPA, Neuromodulation

## Abstract

**Background:**

Predatory stress as a psychological stressor can elicit the activation of the hypothalamic–pituitary–adrenal (HPA) axis, which is involved in the dialogue of the neuroimmunoendocrine network. The brain has been proven to regulate the activity of the HPA axis by way of lateralization. In the present study, we probed the pivotal elements of the HPA circuitry including CRH, GR and a multifunctional cytokine in behavior-lateralized mice to determine their changes when the animals were subjected to predator exposure.

**Methods:**

Behavior-lateralized mice were classified into left-pawed and right-pawed mice through a paw-preference test. Thereafter, mice in the acute stress group received a single 60-min cat exposure, and mice in the chronic group received daily 60-min cat exposure for 14 consecutive days. The plasma CS and TNF-α were determined by ELISA, the hypothalamic CRH mRNA and hippocampal GR mRNA were detected by real-time PCR, and the hippocampal GR protein was detected by western blot analysis.

**Results:**

The results revealed that the levels of plasma CS were significantly elevated after chronic predatory exposure in both right-pawed and left-pawed mice; the right-pawed mice exhibited a higher plasma CS level than the left-pawed mice. Similarly, the acute or chronic cat exposure could induce the release of plasma TNF-α, and the left-pawed mice tended to show a higher level after the acute stress. Chronic stress significantly upregulated the expression of hypothalamic CRH mRNA in both left-pawed and right-pawed mice. Normally, the left-pawed mice exhibited a higher GR expression in the hippocampus than the right-pawed mice. After the cat exposure, the expression of GR in both left-pawed and right-pawed mice was revealed to be greatly downregulated.

**Conclusion:**

Our findings indicate that predatory stress can invoke a differential response of stressful elements in behavior-lateralized mice. Some of these responses shaped by behavioral lateralization might be helpful for facilitating adaption to various stimuli.

## Background

The nature and course of stress can influence an individual’s ability to cope with life events, which might result in various consequences [1–4]. Predatory exposure (exposing rodents to a non-attacking predator or to the predator’s odors) is a commonly used psychological stressor, triggering effects that are similar to human stress-linked phenomena [5–7]. Resembling most types of stimuli, predatory stress elicits increased activity of the hypothalamic–pituitary–adrenal (HPA) axis, which is involved in the dialogue of neuroimmunoendocrine networks. The activity of the HPA axis is modulated by a series of elements in the central loop such as corticotropin releasing hormone (CRH), adrenocorticotrophic hormone (ACTH) and glucocorticoids [8]. Among these, CRH, which has been predominantly detected in the paraventricular nucleus (PVN) of the hypothalamus, plays a potent role in regulating psycho-immunology and behavioral effects to stimuli [9]. Normally, a stressful state might trigger the secretion of CRH from the hypothalamus; in turn, CRH activates the release of ACTH from the pituitary and finally stimulates the release of glucocorticoids from the adrenal cortex. In the circuitry, glucocorticoids are the final effectors to stressful reactions [10, 11]. When binding to the glucocorticoid receptor (GR) [12, 13], glucocorticoids trigger suppressive effects on the release of various inflammation-related chemical mediators such as TNF-α [14]. The related effects stated above can also be regulated through hippocampal structures. As a facilitator in the stress loop, changes in the GR would presumably exert effects on the body’s neuroimmunoendocrine function. Therefore, all stressful elements in the circuitry orchestrate and regulate the basal activity of the HPA axis to maintain homeostasis.

Ascertained by many experiments, the brain modulates neurochemical and functional activity through lateralization. The asymmetric brain is thought to be responsible for the differences of certain cognitive and motor tasks. Behavioral lateralization, which reflects functional brain asymmetry, is a commonly-existing phenomenon. Recent research provides evidences for communication between brain lateralization and the immune system. A good case is that brain lateralized-animals exhibit various responses to environmental stimuli at both the individual level and the population level. In mice, based on paw preference model as an index of asymmetry, it has been confirmed that there were notable relationships between immune parameters and behavioral lateralization. The immune parameters including natural killer cell activity, cytotoxic T lymphocyte activity, mitogen-induced lymphoproliferation show an association with paw preference [15–18]. The plasma levels of IL-1 and IL-6 are also different in various strains of left-pawed and right-pawed mice [19, 20]. The normal left-pawed BALB/c mice had higher plasma levels of IL-1 than the right-pawed mice. Additionally, male BALB/c mice displayed a link between turning preference with host against *Listeria monocytogenes* and especially with the production of IL-6 and interferon-gamma (IFN-Ɣ) [19, 20]. Besides, as for the stress system, responses including the activation of HPA axis, secretion of glucocorticoids and cytokines from the cortex, hypothalamus and hippocampus, have been proven to exhibit great differences in behavior asymmetric groups under physiological conditions, LPS-treatment or stressful conditions [21, 22]. The involved mediators above even showed a correspondence with the direction and intensity of behavioral lateralization [22]. The activity of the HPA axis is governed by key elements of stress circuitry in the crosstalk of the neuroimmunoendocrine network. Although the brain could modulate HPA activity asymmetrically, resulting in various alterations in corticosterone (CS) levels for the left-handed/left-pawed and right-handed/right-pawed groups, it remains unclear whether the key elements of HPA circuitry are involved in the regulation of behavior lateralization in response to psychological stressors. Hence, in the present study, we probed the pivotal elements of HPA circuitry including the CRH, GR and a multifunctional cytokine in behavior-lateralized mice, to determine their changes when the animals were subjected to predator exposure.

## Methods

### Animals

Female BALB/cAnN mice (4 weeks old) were used in our studies. Two short-haired male cats (6 months old; body weight 1.7 and 2.0 kg) were utilized as the predator stimulus. The mice and the cats were adapted to the environment before any behavioral experiments. All efforts were taken to minimize the number of animals and their suffering. All animal experiments were approved by the Animal Care and Use Committee of Shantou University Medical College and were carried out in compliance with the National Institutes of Health Guide for the Care and Use of Laboratory Animals.

### Behavior lateralization test

The paw-preference test was used for the classification of the behavior-lateralized animals [19]. The mice were deprived of food for 18 h overnight and were then kept in a testing cubicle with a feeding tube. The mice could reach a pellet of food only with the use of one paw. The numbers of right-paw entries (RPE) per 50 paw reaches to reach the food pellet were scored. Over a 2-week period, the mice were tested for four sessions. Based on the RPE score, the animals were classified as left-pawed mice if the RPE score was equal to or less than 20 or were classified as right-pawed mice if the RPE score was equal to or above 30. Twenty-four right-pawed and 24 left-pawed mice were used in our experiments.

### Predatory stress procedures

The right-pawed and left-pawed mice were divided into the unstressed control group (normal control group), acute stress group and chronic stress group (each group was composed of eight mice). The acute stress group received a single 60-min cat exposure, and the chronic stress group received daily 60-min cat exposure for 14 consecutive days. The cat-exposed mice were first placed into a small cage, which allowed for the mice to have access to the visual, olfactory, and acoustic stimuli associated with the cats but prohibited physical interaction or a real attack. Next, the cage was placed in a larger compartment with the cats. Observation was conducted from outside the room without interference. After completing the cat test, all mice were sacrificed by rapid decapitation. Trunk blood samples and the hippocampus and hypothalamus of the brain were collected quickly and stored for further processing.

### Detection of CS and TNF-α

The levels of plasma CS and TNF-α were detected via the enzyme-linked immunosorbent assay (CS, TNF-α ELISA kits, BD, USA). All procedures were performed according to the manufacturer’s protocols.

### Real-time PCR

The total RNA from the hippocampus and hypothalamus was extracted using TRIzol^**®**^ reagent (Invitrogen, USA). For the synthesis of cDNA, the total RNA was reversely transcribed using Moloney murine leukemia virus reverse transcriptase (Invitrogen, USA). For quantitative real-time PCR, SYBR Green Supermix (Invitrogen, USA) was used for the detection of the CRH mRNA and GR mRNA. The sequences of primers specific for CRH, GR and glyceraldehyde-3-phosphate dehydrogenase (GAPDH) were listed as follows (Table 1). All reactions were performed in triplicate. ABI Prism7300 was applied for analysis. GAPDH was used as the endogenous reference gene. Each sample for CRH and GR gene was calculated using the 2^−∆∆Ct^ method. The normal control group (not considering the effects of the behavior lateralization) was chosen as the calibrator for normalization. Changes in relative expression of CRH mRNA and GR mRNA were evaluated as sample/normal control.

**Table 1:** Specific primers used in real-time PCR analysis

Gene	Forward	Reverse
CRH	5′CACCTACCAAGGGAGGAGAA 3′	5′CAGAGCCACCAGCAGCAT 3′
GR	5′ATGGGCAAAGGCGATACCAGGATT 3′	5′CCAACCCAGGGCAAATGCCATGA 3
GAPDH	5′GTGACTTCAACAGCAACTCCCATT3′	5′GTTATGGGGTCTGGGATGGAATTGTG3′

### Western blotting

The total protein concentration was determined using the BCA protein assay kit (Beyotime, China). The protein samples were subjected to electrophoresis via SDS-PAGE and were transferred onto a PVDF membrane (Roche, UK) using a blot system, according to standard protocols. Antibodies against GR (Santa Cruz, USA) and β-actin (Beyotime, China) and a secondary antibody conjugated to horseradish peroxidase (Beyotime, China) were used for image detection. The images were visualized by Western Blotting Luminol Reagent (Santa Cruz, USA) via Gel Imaging System (Alpha Innotech, US). The band intensity was determined using the ImageJ software. The relative ratio (GR/β-actin) was calculated.

### Statistical analyses

An analysis of variance (ANOVA) was performed after verifying a normal distribution of data and the equality of variances. Log transformation or square root transformation was performed when the normal test failed.

In the presence of behavioral lateralization in mice: statistical analysis of the CS and TNF-α levels in the plasma, the CRH mRNA expression in the hypothalamus, and GR expression in the hippocampus was conducted by two-way ANOVA with stress as a between-groups factor (three levels: normal control, acute stress, and chronic stress) and behavioral lateralization as a between-groups factor (two levels: left-pawed and right-pawed). This was followed by the post hoc test (Bonferroni correction). The *t*-test was used to analyze differences between the right-pawed and left-pawed mice in each group.

In the absence of behavioral lateralization in mice: the data regarding the plasma CS and TNF levels, the hypothalamic expression of the CRH mRNA, and the hippocampal expression of the GR were analyzed by one-way ANOVA with stress as a between-groups factor with 3 levels (normal control, acute stress, and chronic stress). The post hoc test (Bonferroni test) was performed to determine the difference between each group when the stress effect was found. A *t*-test was performed to analyze the differences between the right-pawed and left-pawed mice. Statistical analysis was performed by using SPSS 19.0 and Microsoft Excel software. The data were expressed as the mean ± SEM. A P value < 0.05 was considered statistically significant.

## Results

### The levels of plasma CS in behavior-lateralized mice after predatory exposure

**Fig. 1:**
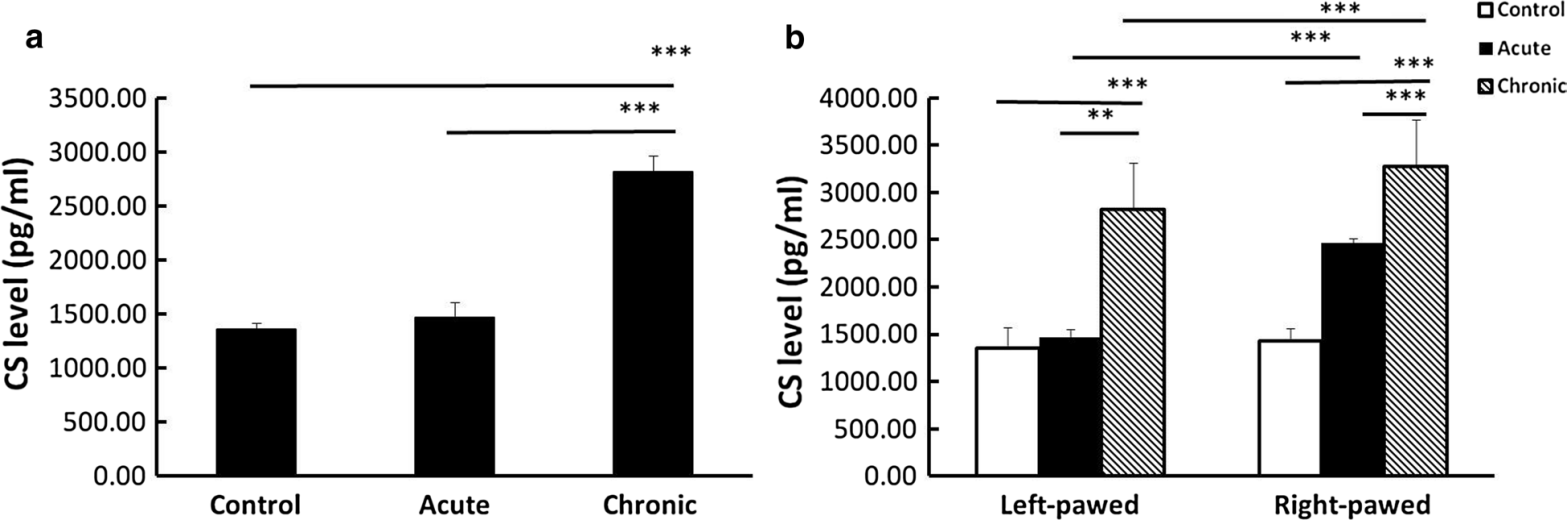
The levels of plasma CS in behavior-lateralized mice after predatory stress (8 mice in each group). **a** Influence of predator exposure on plasma CS. The chronic predatory stress increased the plasma CS to a higher extent than that in the control and acute stress groups (*P < 0.05, **P < 0.01, ***P < 0.001). **b** Plasma CS in behavior-lateralized mice with predatory stress. The right-pawed mice showed a higher plasma CS concentration than the left-pawed mice after acute stress and chronic stress. Significant differences were observed between the left-pawed and right-pawed mice in each group (*t*-test) (*P < 0.05, **P < 0.01, ***P < 0.001)

Figure 1 shows the plasma CS level in behavior-lateralized mice after predatory exposure. A two-way ANOVA revealed a significant predatory stress effect [*F*(2,42) = 86.26; P < 0.001], a brain lateralization effect [*F*(1,42) = 47.11; P < 0.001], and a stress × lateralization interaction [*F*(2,42) = 10.56; P < 0.01]. The plasma CS level were elevated after chronic predatory exposure, exhibiting a higher level than that in the normal control (P < 0.001) and acute stress groups (P < 0.001) (Fig. 1a). When the effect of behavioral lateralization was considered, the CS level increased after chronic predatory stress in both right-pawed and left-pawed mice (Fig. 1b). The right-pawed mice exhibited a higher plasma CS concentration than the left-pawed mice after acute (P < 0.001) and chronic stress (P < 0.001).

### The levels of plasma TNF-α in behavior-lateralized mice after predatory exposure

**Fig. 2:**
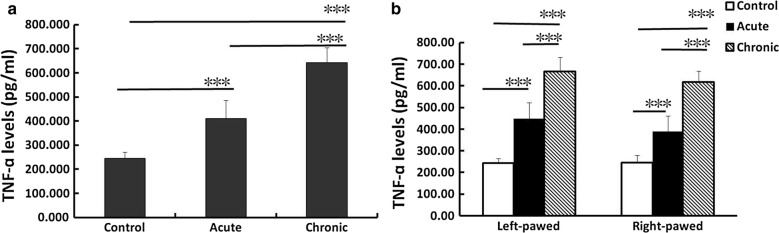
The levels of plasma TNF-α in behavior-lateralized mice after predatory stress (8 mice in each group). **a** Influence of predator exposure on plasma TNF-α. The TNF-α level of the acute and chronic stress groups were significantly elevated (*P < 0.05, **P < 0.01, ***P < 0.001). **b** Plasma TNF-α in behavior-lateralized mice with predatory stress. The left-pawed and right-pawed mice showed significantly elevated TNF-α levels after acute and chronic predatory stress (*P < 0.05, **P < 0.01, ***P < 0.001)

The levels of plasma TNF-α are shown in Fig. 2. The analysis demonstrated a significant effect of predatory stress [*F*(2,42) = 197.13; P < 0.001] but no effects of behavioral lateralization [*F*(1,42) = 3.50; P = 0.068] and stress × lateralization interaction [*F*(2,42) = 0.934; P = 0.401]. One-way ANOVA revealed that the TNF-α levels were significantly elevated in the acute (P < 0.001) and chronic stress groups (P < 0.001) (Fig. 2a), especially in the chronic stress mice. The left-pawed and right-pawed mice showed increased TNF-α levels after acute stress (P < 0.001) and chronic stress (P < 0.001) (Fig. 2b). The left-pawed mice tended to exhibit higher levels of TNF-α than the right-pawed mice after acute (P = 0.12) and chronic stress (P = 0.099); however, no significant differences were observed.

### The expression of CRH mRNA in the hypothalamus of behavior-lateralized mice after predator exposure

**Fig. 3:**
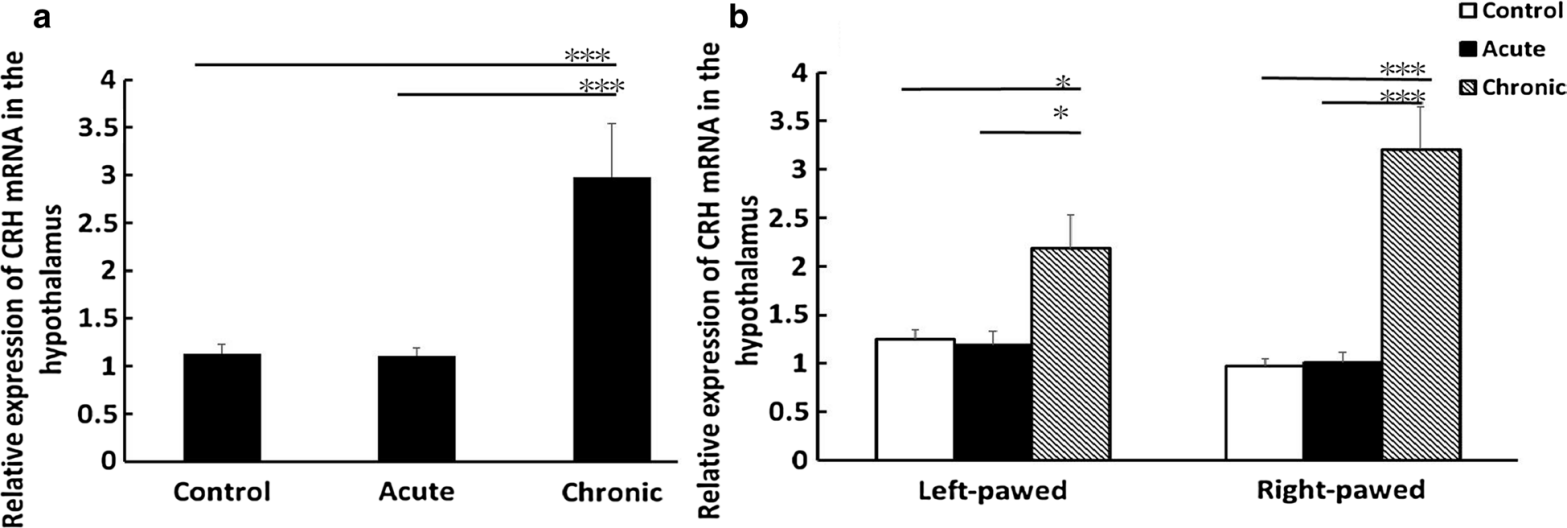
The expression of CRH mRNA in the hypothalamus of behavior-lateralized mice after predatory exposure (8 mice in each group). **a** Influence of predator exposure on the expression of CRH mRNA in the hypothalamus. Chronic predator stress could significantly increase CRH expression. The normal control group (without considering behavioral lateralization) was used as the calibrator for normalization (*P < 0.05, **P < 0.01, ***P < 0.001). **b** The expression of CRH mRNA in the hypothalamus of behavior-lateralized mice with predatory stress. Both in the left-pawed and right-pawed mice, CRH mRNA expression was greatly elevated after chronic stress, showing higher levels than those in the control and acute stress mice. The normal control group (without considering behavioral lateralization) was used as the calibrator for normalization (*P < 0.05, **P < 0.01, ***P < 0.001)

Considering stress and behavioral lateralization (right-pawed and left-pawed) as factors, two-way ANOVA showed only a stress effect [*F*(2,42) = 28.75, P < 0.001] and stress × lateralization interaction [*F*(2,42) = 4.417; P = 0.023], but no lateralization effect [*F*(1,42) = 0.90, P = 0.351] on the expression of the CRH mRNA in the hypothalamus. Considering the normal control group as the calibrator, our real-time PCR data revealed that the chronic predator stress increased the expression of the CRH mRNA when compared with the control group (P < 0.001) and the acute stress group (P < 0.001) (Fig. 3a). Both in the left-pawed and right-pawed mice, the expression of CRH mRNA was profoundly elevated after chronic stress, when compared with the control (left-pawed: chronic stress vs control, P = 0.035; right-pawed: chronic stress vs control, P < 0.001) and acute stress groups (left-pawed: chronic stress vs acute stress, P = 0.022; right-pawed: chronic stress vs acute stress, P < 0.001) (Fig. 3b).

### The expression of GR in the hippocampus of behavior-lateralized mice after predatory exposure

**Fig. 4:**
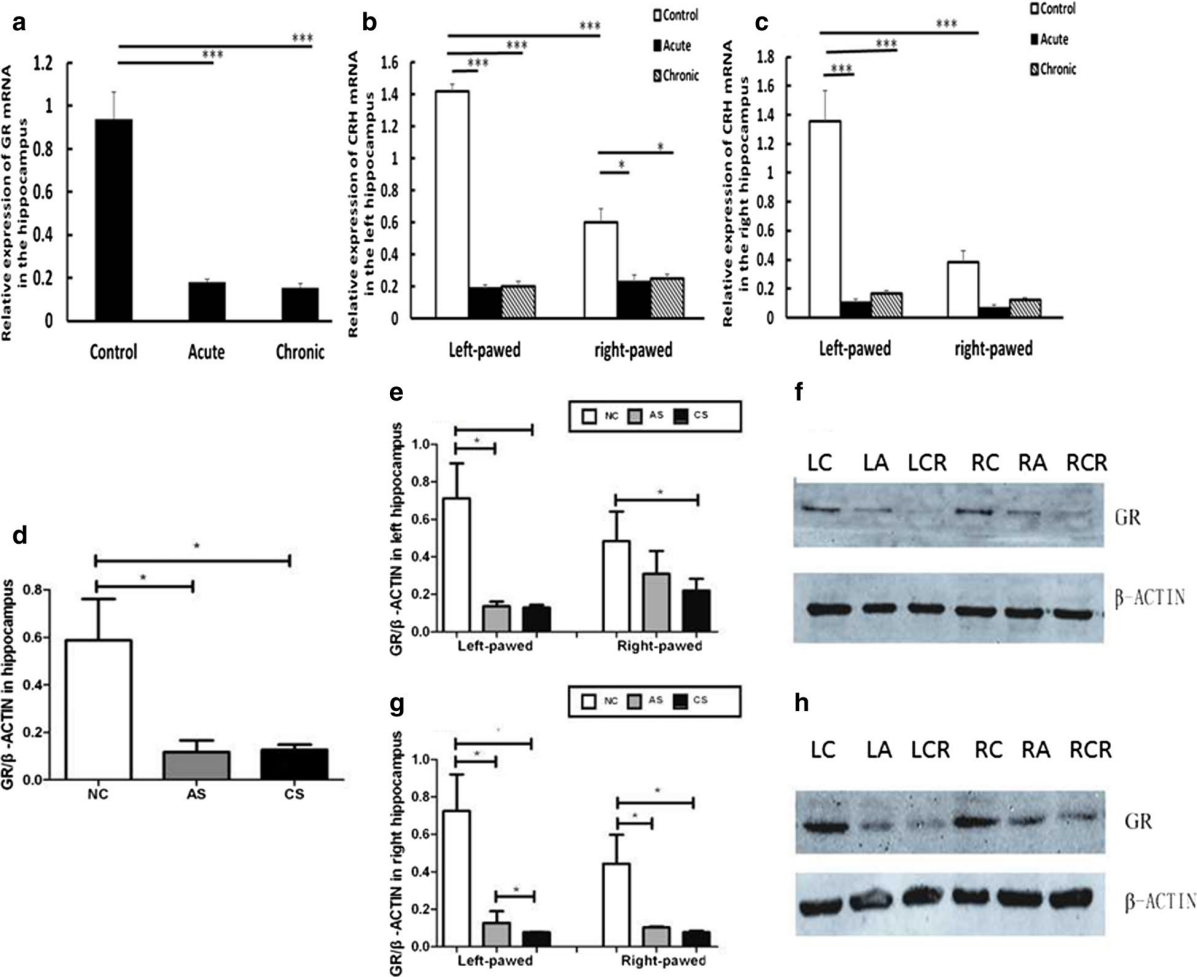
The expression of GR in the hippocampus of behavior-lateralized mice after predatory exposure (8 mice in each group). **a** Influence of predatory exposure on the expression of GR mRNA. The GR mRNA expression in the hippocampus was downregulated profoundly after acute stress and chronic stress. The normal control group (without considering behavioral lateralization) was used as the calibrator for normalization (*P < 0.05, **P < 0.01, ***P < 0.001). **b**, **c** The expression of GR mRNA in the hippocampus of behavior-lateralized mice after predatory stress. Acute and chronic stress reduced the GR mRNA expression significantly, particularly in the left-pawed mice. For the normal control group, the GR mRNA in the left and right hippocampus of the left-pawed mice was higher than that of the right-pawed mice. The normal control group (without considering behavioral lateralization) was used as the calibrator for normalization (*P < 0.05, **P < 0.01, ***P < 0.001). **d**–**h** The expression of the GR protein in the hippocampus of behavior-lateralized mice after predatory exposure. The acute and chronic stress decreased the GR protein expression in the left and right hippocampus of both right-pawed and left-pawed mice (relative density: *P < 0.05). *Lc* left-pawed mice in control group, *Las* left-pawed mice in acute stress group, *Lcs* left-pawed mice in chronic stress group, *Rc* right-pawed mice in control group, *Ras* right-pawed mice in acute stress group, *Rcs* right-pawed mice in chronic stress group

For the GR mRNA, one-way ANOVA demonstrated that acute and chronic predatory stress could downregulate the expression after acute (P < 0.001) and chronic stress (P < 0.001) (Fig. 4a). Two-way ANOVA showed a predatory stress effect [*F*(2,42) = 59.04, P < 0.001], a brain lateralization effect [*F*(1,42) = 12.45, P = 0.002], and a stress × lateralization interaction [*F*(2,42) = 17.71, P < 0.001] in the left hippocampus. Similarly, a predatory stress effect [*F*(2,42) = 43.17, P < 0.001], a brain lateralization effect [*F*(1,42) = 21.04, P < 0.001], and a stress × lateralization interaction [*F*(2,42) = 16.42, P < 0.001] were also found in the right hippocampus. The data showed that the normal control group exhibited a higher expression of the GR mRNA in the left (P < 0.001) and right hippocampus (P < 0.001) of the left-pawed mice than that in the right-pawed mice (Fig. 4b, c). After stress, the expression of GR in the left-pawed mice was downregulated more profoundly than that in the right-pawed mice. Western blot analysis revealed that the changes in the GR protein were similar to those in the GR mRNA expression when considering stress as a factor. The expression of the GR protein was reduced both in the left and right hippocampal formation of the acute (P < 0.05) and chronic stress groups (P < 0.05) (Fig. 4d–h).

## Discussion

The activation of the stress system might lead to behavioral and immunoendocrine changes, which can improve the ability to adjust to homeostasis [23, 24]. In the present study, the acute and chronic cat-exposure model was chosen to act as a psychological stressor to induce internal stress responses, and the impact on neural and hormonal indices was evaluated. Our results indicated that predator stress engaged an activation of the HPA axis that might fuel long-lasting changes in brain stress. Generally, the stressful response with the activation of the HPA axis is acute or at least of a limited duration. Our data suggested that the integrated CS response was sensitized by repeated predator exposure. When taking behavioral lateralization into account, the right-pawed mice exhibited a higher elevation in the CS concentration after the acute and chronic predator exposure than the left-pawed mice. The elevation of CS in the behavior-lateralized mice may be related to the changes of the other elements in the stress circuitry such as GR or CRH, which eventually elicit the negative feedback regulation of the HPA axis. The increased CS after stress had vital significance. It could regulate inflammation and use the body’s energy metabolism to deal with an emergent situation such as survival from predators. TNF-α is a multifunctional mediator, which can modulate the neuroimmunoendocrine system. Generally, a moderate plasma TNF-α level can maintain the immune activation and inflammatory reaction. The results showed that the plasma TNF-α was significantly elevated after acute and chronic stress, and the chronic stress group showed a higher TNF-α level than the acute stress group. These findings implied that psychological stress might trigger immune activity responsively. When we considered the effects of behavioral asymmetry, TNF-α was increased in both left-pawed and right-pawed mice after the predatory stress, and the left-pawed mice tended to exhibit a high concentration of TNF-α. Such changes suggested that behavior lateralization may exert effects on the release of cytokines from immune cells to a certain extent, and the behavior-lateralized mice may react to psychological stimulus heterogeneously. Previous studies have found that left-pawed BALB/c mice exhibited a higher plasma IL-1β level and a higher activity of the HPA axis physiologically [15, 25]. Plasma IL-6 and ACTH were also involved in the regulation of behavioral lateralization with the challenge of LPS [16, 19, 21, 25]. Hence, overall, when confronting various stressors including psychological stimuli or infection, distinct groups with hand/paw preference may adjust their sensitivity of distinct stressful components coordinately, so that they can facilitate internal homeostasis.

The hippocampal and hypothalamic regions have been proven to exhibit high densities of adrenocorticosteroid-binding sites within the brain, which exert pivot feedback effects on the HPA axis [13, 16, 26]. Commonly, a stimulus can result in higher hormone secretion and activity of the HPA axis, which are always involved in the negative feedback regulation of immune functions. According to our experimental design, the alteration of the HPA axis could be determined in a naturalistic manner. In the current study, the CRH mRNA expression in the hypothalamus elevated after acute and chronic stress, particularly after chronic stress. It appeared that such variation of the CRH mRNA in this region was necessary for inducing facilitation of the HPA loop [11, 26–28]. Furthermore, both left-pawed and right-pawed mice exhibited elevation in the CRH expression after chronic predator exposure, and the left-pawed mice were inclined to exhibit a lower level than the right-pawed mice. These phenomena may be ascribed to genetic differences in vulnerability to stress and the rate of steroid degradation in behavior asymmetric animals. Previous data demonstrated that the left-pawed mice have lower HPA-axis reactivity; this alteration might be related to the consistent alteration of CRH expression. Therefore, with lower activity of the stress circuitry, the left-pawed mice may display lower anti-inflammatory effects to stressful sufferings than the right-pawed mice in the short-duration or long-time pathological conditions. These clues supplied further evidence to prove the previous reports that left-pawed mice might be more susceptible to immune disorders or certain brain disorders than right-pawed mice, and to explain partially why left-paw preference or left-handedness is associated with a higher incidence of immune-mediated disorders [29–31]. In addition, GR is also a critical anti-inflammatory molecule in the central stress circuitry [14]. Studies demonstrated that GR could suppress synthesis and release of some inflammatory chemical mediators and cytokines [14]. The present study showed that the GR expression in the hippocampus of the control left-pawed mice was higher than that of the right-pawed control, while the GR expression in the hippocampus of the left-pawed mice was downregulated profoundly after acute and chronic predator exposure. The downregulation of the GR expression might lead to decrease of its function, especially its anti-inflammatory capacity in left-pawed mice.

## Conclusion

Taken together, the findings of our study indicated that predatory exposure can invoke heterogeneous variation of the stressful elements of the neuroimmunoendocrine central circuitry, which could promote complicated responses and sensitization of the HPA axis in behavioral-lateralized animals. Due to these factors, the behavior-lateralized animals showed differential responses to psychological stressors, which would be beneficial in adapting to dynamic environments.

## Abbreviations

ACTH: adrenocorticotrophic hormone
CRH: corticotrophic hormone-releasing hormone
CS: corticosterone
ELISA: enzyme linked immunosorbent assay
GR: glucocorticoid receptor
HPA: hypothalamic–pituitary–adrenal axis
IL-1β: interleukin-1β
IL-6: interleukin-6
IFN-Ɣ: interferon-gamma
PVN: paraventricular nucleus
RPE: right paw entries
TNF-α: tumor necrosis factor-α
